# Structural Modeling and Molecular Dynamics of the Immune Checkpoint Molecule HLA-G

**DOI:** 10.3389/fimmu.2020.575076

**Published:** 2020-11-06

**Authors:** Thais Arns, Dinler A. Antunes, Jayvee R. Abella, Maurício M. Rigo, Lydia E. Kavraki, Silvana Giuliatti, Eduardo A. Donadi

**Affiliations:** ^1^ Department of Basic and Applied Immunology, Ribeirão Preto Medical School, University of São Paulo, Ribeirão Preto, Brazil; ^2^ Department of Computer Science, Rice University, Houston, TX, United States; ^3^ Department of Genetics, Ribeirão Preto Medical School, University of São Paulo, Ribeirão Preto, Brazil

**Keywords:** HLA-G, HLA-G1 soluble dimer, HLA-G5 isoform, molecular dynamics, structural bioinformatics

## Abstract

HLA-G is considered to be an immune checkpoint molecule, a function that is closely linked to the structure and dynamics of the different HLA-G isoforms. Unfortunately, little is known about the structure and dynamics of these isoforms. For instance, there are only seven crystal structures of HLA-G molecules, being all related to a single isoform, and in some cases lacking important residues associated to the interaction with leukocyte receptors. In addition, they lack information on the dynamics of both membrane-bound HLA-G forms, and soluble forms. We took advantage of *in silico* strategies to disclose the dynamic behavior of selected HLA-G forms, including the membrane-bound HLA-G1 molecule, soluble HLA-G1 dimer, and HLA-G5 isoform. Both the membrane-bound HLA-G1 molecule and the soluble HLA-G1 dimer were quite stable. Residues involved in the interaction with ILT2 and ILT4 receptors (α3 domain) were very close to the lipid bilayer in the complete HLA-G1 molecule, which might limit accessibility. On the other hand, these residues can be completely exposed in the soluble HLA-G1 dimer, due to the free rotation of the disulfide bridge (Cys42/Cys42). In fact, we speculate that this free rotation of each protomer (i.e., the chains composing the dimer) could enable alternative binding modes for ILT2/ILT4 receptors, which in turn could be associated with greater affinity of the soluble HLA-G1 dimer. Structural analysis of the HLA-G5 isoform demonstrated higher stability for the complex containing the peptide and coupled β2-microglobulin, while structures lacking such domains were significantly unstable. This study reports for the first time structural conformations for the HLA-G5 isoform and the dynamic behavior of HLA-G1 molecules under simulated biological conditions. All modeled structures were made available through GitHub (https://github.com/KavrakiLab/), enabling their use as templates for modeling other alleles and isoforms, as well as for other computational analyses to investigate key molecular interactions.

## Introduction

The Human Leukocyte Antigen G (HLA-G) is a nonclassical Major Histocompatibility Complex class I (MHC-I) molecule that possesses immunomodulatory properties (1). Its presence is tissue-restricted, being expressed in fetal tissues [trophoblast cells (2)] and constitutively expressed in adult thymic medulla (3), cornea (4), pancreatic islets (5), erythroid, and endothelial cell precursors (6). However, the expression of HLA-G can be induced in several conditions (1), including cancer (7, 8), transplantation (9), viral infections (10, 11), and autoimmune and inflammatory diseases (12, 13).

A well-recognized function of the HLA-G molecule in these pathological and physiological conditions is the inhibition of the cytotoxic activity of Natural Killer (NK) and CD8^+^ T lymphocytes. This function is mediated by interaction with leukocyte receptors, particularly with the Leukocyte Ig-like Receptors (LILRs), also known as Immunoglobulin-like Transcripts (ILT2, ILT4). ILT2 and ILT4 interact with several classical class I HLA molecules, but have higher affinity for HLA-G (14). ILT2 is expressed by B cells, some subtypes of T cells and NK cells, and all monocytes/dendritic cells (15). It is also described as a receptor for HLA-G associated with β2-microglobulin. On the other hand, ILT4 is myeloid-specific and only expressed by monocytes/dendritic cells (16), being capable of recognizing HLA-G free heavy chains (17, 18). Through these differentially expressed receptors, HLA-G can interact with all these different cell types, primarily inhibiting their functions. In addition, HLA-G may also generate regulatory/suppressor cells. For instance, human tolerogenic dendritic cells (DC-10) express high levels of membrane-bound HLA-G1 and are potent inducers of adaptive allospecific Type 1 regulatory T (Tr1) cells (19). The *HLA-G* gene is located within the MHC region, presenting low polymorphism, in contrast with the highly polymorphic classical class I genes, i.e., *HLA-A*, *-B*, *-C* (20). Geragthy et al. (21) first described the *HLA-G* gene in 1987, and its structure is homologous to other HLA class I genes. The *HLA-G* primary transcript may generate at least seven alternative splicing mRNAs that encode membrane-bound (HLA-G1, G2, G3, G4) and soluble (HLA-G5, G6, G7) protein isoforms (22–25). HLA-G1 may also be detected in plasma after proteolytic cleavage by metalloproteases, and presents the same domains (α1, α2, and α3) of classical class I molecules, being also associated with a β2-microglobulin. HLA-G2 is devoid of the α2 domain encoded by exon 3. HLA-G3 does not have the α2 and α3 domains encoded by exons 3 and 4, and HLA-G4 lost the α3 domain. The soluble HLA-G5 and HLA-G6 isoforms have the same extra globular domains as HLA-G1 and HLA-G2, respectively, and are generated by transcripts retaining intron 4, which block translation of the transmembrane domain (exon 5). The 5’ region of the intron, in the reading phase with exon 4, is translated into a stop codon and generates the HLA-G5 and HLA-G6 isoforms. These isoforms contain a specific 21 residues long tail involved in molecule solubility. The soluble HLA-G7 isoform is limited to the α1 domain and retains two intron 2 specific amino acids. All alternative transcripts are devoid of exon 7 (26, 27).

Sequence comparison of the HLA-G molecule to other HLA class I proteins reveals some interesting particularities. First, HLA-G has an unusually long half-life on the cell surface, resulting from the absence of an endocytosis motif in its truncated cytoplasmic domain (28). Second, HLA-G sequences have two unique Cysteine residues located at positions 42 and 147. Dimerization of HLA-G occurs through the creation of disulfide bonds between the two unique Cysteine residues at position 42 (Cys42-Cys42 bonds). Since all isoforms carry Cys42, all translated isoforms could potentially form membrane-bound homodimers, soluble homodimers, β2-microglobulin-free homodimers, and possibly homotrimers (associated or not to β2-microglobulin) (29, 30). Noteworthy, HLA-G dimers: *i)* do not induce significant structural changes to the main backbone of the protomers (i.e., chains forming the dimer) (17); *ii)* may exhibit distinct inhibitory functions as compared to monomers [e.g., dimers bind to ILT receptors with higher affinity *in vitro* (29) and *in vivo* (31)]; and *iii)* exhibit slower dissociation rates than monomers (17). ILT recognition of HLA-G dimers has a pivotal role on immune suppression at the maternal-fetal interface, possibly contributing to the prevention of pregnancy complications such as pre-eclampsia and recurrent miscarriages (17, 20).

Since HLA-G5 isoform has the same extra globular domains as HLA-G1, it could potentially be recognized by the same receptors. In fact, it has been reported that ILT2 can interact with β2-microglobulin-associated HLA-G5, while ILT4 could be able to recognize isoforms that are not associated to β2-microglobulin (17, 32). Such β2-microglobulin-free heavy chain has been detected in cell culture supernatants expressing HLA-G5 (33). It has also been shown that the expression of soluble HLA-G5 could inhibit the cytotoxicity of NK cells, and that the degree of inhibition was more evident when induced by HLA-G5, as compared to the membrane-bound HLA-G1. Most importantly, it was shown that the combination of HLA-G1 and HLA-G5 leads to significantly greater suppression than the effects of HLA-G1 or HLA-G5 alone (34). The direct involvement of HLA-G5 in inducing graft acceptance *in vivo* after human transplantation was provided by the observation that HLA-G5 purified from the plasma of transplanted HLA-G-positive patients suppressed alloproliferation of T cells *in vitro* (35).

Considering all the aforementioned structural diversity of known HLA-G isoforms, and the multiple roles of HLA-G in different immunological pathways, it is astonishing how little is known about the structure and dynamics of these molecules. As of today, there are only seven crystal structures of HLA-G receptors in the Protein Data Bank (PDB) (36). Note that these structures are limited to HLA-G1, and that even for this particular isoform they do not capture the full molecule (see **Supplementary Table 1**). In addition, there is only so much that can be understood from a static crystal structure in which relates to the dynamic behavior of these molecules. For instance, previous analysis of the membrane-bound HLA-G1 has indicated an oblique orientation of the protomers. Such orientation makes the ILT2 and ILT4 binding sites slightly more accessible to the interaction with these receptors (17). However, it does not tell us if this oblique orientation is stable in the soluble HLA-G1 dimer, or if other arrangements are possible. Finally, available structural data cannot inform us about the structure and dynamics of all other HLA-G alleles and isoforms.

**Figure 1:**
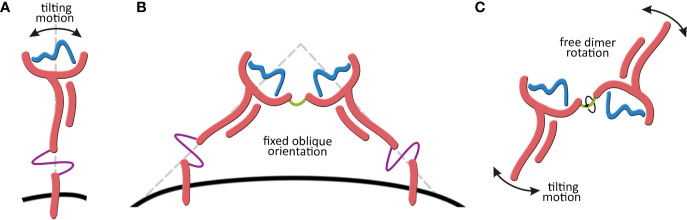
**(A)** Tilting motion of the membrane-bound HLA-G1structure. **(B)** Membrane-bound HLA-G1 dimer representation, showing the oblique orientation (~45° angle) observed by X-ray crystallography. **(C)** Representation of the complete rotational freedom of the soluble HLA-G1 dimer in solution.

As a step forward in addressing all these open questions, the present work reports for the first time the complete structure and dynamic behavior of the membrane-bound HLA-G1 model. In addition, it also characterizes the dynamics of the soluble HLA-G1 dimer. These efforts allowed for the first time the observation of a tilting movement of the membrane-bound HLA-G1 monomer, and the total rotational freedom of the HLA-G1 dimer in solution (**Figure 1**). Finally, it investigates the stability of three different proposed structures for the soluble HLA-G5 isoform.

## Material and Methods

### Molecular Modeling

To obtain the complete HLA-G1 model for the molecule encoded by the HLA-G*01:01 allele group, homology modeling was performed using Modeller 9.15 software (37) and the PDB_ID: 1YDP structure as a template (38). The selected template structure was obtained by X-ray diffraction crystallography with a 1.9 Å resolution (38), is encoded by the HLA-G*01:04 allele group, and exhibits 275 resolved residues. It includes the nonapeptide RIIPRHLQL in the binding cleft, and the coupled β2-microglobulin chain. The Rosetta cyclic coordinate descent algorithm (CCD) *ab initio* modeling (39) was applied to unresolved extracellular and intracellular regions in the crystallographic template. Two thousand models were generated in each *ab initio* modeling step. For the transmembrane portion, the GPCR-ITASSER online server was used (40). The complete membrane-bound HLA-G1 model was then applied as template for three possible HLA-G5 isoform structures: monomer, monomer containing the nonapeptide in the cleft, and monomer containing the nonapeptide in the cleft coupled to β2-microglobulin. Isoform residues not included in the membrane-bound HLA-G1 model were resolved using the Rosetta CCD *ab initio* modeling. The existing structural gaps in the HLA-G1 soluble dimer template (PDB_ID: 2D31) were completed by homology modeling using PDB_ID: 1YDP structure as template. All models were evaluated using several validation software, including QMEAN (41), MODFOLD (42), Verify 3D (43, 44), ERRAT (45), and PROCHECK (46). Images and structure visualization were performed using PyMOL software (47). The BioPython package (48) was applied to identify the interacting residues. The Cα Root Mean Square Deviation (RMSD) and Root Mean Square Fluctuations (RMSF) values were calculated using the initial structures as reference. All structures and simulation movies are available in the **Supplementary Material** and at GitHub (https://github.com/KavrakiLab/).

### Lipid Bilayer Insertion

The complete HLA-G1 model was inserted into a phospholipid bilayer (DLPA, *1,2-Dilauroyl-sn-glycero-3-phosphate*). This step was performed with the CHARMM-GUI online server (49, 50).

### Molecular Dynamics (MD) Simulations

Three simulations of 100 ns were performed for the complete HLA-G1 inserted into the lipid bilayer, using GROMACS v5.1.4 (51) and CHARMM36m force field (52). MD simulations were also performed in triplicate using GROMACS v4.6.5 package and the G54a7 force field, for a total of 600 ns for the soluble HLA-G1 dimer and a total of 2.1 μs for the HLA-G5 isoform. A cubic box was defined with at least 9 Å of liquid layer around the protein (exact dimensions were different for each protein), using single-point charge water model and periodic boundary conditions. An appropriate number of sodium (Na^+^) and chloride (Cl^−^) counter-ions were added to neutralize the system at the final concentration of 0.15 mol/L. Besides the complete membrane-bound HLA-G1, the dynamic system contained 32,560 DLPA molecules, 184,197 water molecules and 380 counter-ions. As for the soluble dimer, it contained 383,325 water molecules and 470 counter-ions. The HLA-G5 monomer dynamic system contained 90,375 water molecules and 187 counter-ions; the monomer containing the nonapeptide in the cleft system had 90,135 water molecules and 185 counter-ions; and the monomer containing the nonapeptide in the cleft coupled to β2-microglobulin had 83,676 water molecules and 179 counter-ions. The algorithms *v-rescale* (τ*t* = 0.1 ps) and *parrinello-rhaman* (τ*p* = 2 ps) were used for temperature and pressure coupling, respectively. Cutoff values of 1.2 nm were used for both van der Waals and Coulomb interactions, with Fast Particle-Mesh Ewald (PME) electrostatics. For all MD simulations, the production stage was preceded by: *i)* three steps of Energy Minimization (alternating steepest-descent and conjugate gradient algorithms), and *ii)* eight steps of Equilibration as previously described (53). Briefly, the Equilibration stage started with position restraints for all heavy atoms (5,000 kJ^−1^mol^−1^nm^−1^) and a temperature of 310 K, for a period of 300 ps, to allow for the formation of solvation layers. The temperature was then reduced to 280 K and the position restraints were gradually reduced. This process was followed by a gradual increase in temperature (up to 300 K). Together, these Equilibration steps represented the first 500 ps of each simulation. During the production stage, the system was held at constant temperature (310 K) without restraints.

### Dimensionality Reduction Analysis

Principal component analysis (PCA) was performed using the Python libraries MDTraj (54) and PyEmma (55). PCA is a dimensionality reduction method used to analyze the sampling done by the MDs. PCA maximizes the variance of the transformed coordinates, which is ideal for finding conformations that are geometrically diverse. The residue-residue distances (defined as the distance between the nearest two heavy atoms) between one copy of the dimer and the other were extracted. Only every tenth residue in the system was considered to save memory, resulting in 1,444 features for the dimensionality reduction analysis.

### Peptide-Bound Ensemble Modeling and Stability Analysis

A structure-based stability analysis was performed to compare two different HLA-G binders, RIIPRHLQL and RLPKDFRIL. The aforementioned complete model of HLA-G1 (HLA-G*01:01), after removed the bound peptide structure, was used as input to the Anchored Peptide-MHC Ensemble Generator (APE-Gen) (56). Generated ensembles of peptide conformations were later minimized with OpenMM (57), and the lowest energy conformation for each peptide was selected using the Vinardo scoring function (58). All these steps were performed using a customized workflow from the HLA-Arena modeling environment (59). Finally, selected conformations (i.e., lowest energy) were used as input for a structure-based random forest classifier trained on a large dataset of immunopeptidomics experiments (60). This analysis predicted the stability of both complexes, and the individual contribution of each peptide residue toward peptide-MHC complex stability.

### Protein-Protein Docking With ILT4

A protein-protein docking study was conducted with the ClusPro webserver (61). A crystal structure of ILT4 was obtained from PDB (PDB_ID: 6AED), and gaps (residues 134 to 143) were filled with loop refinement algorithm from Modeller 9.15 software (37) using UCSF Chimera software (62). This structure was used for protein-protein docking against *(i)* HLA-G monomer and *(ii)* HLA-G dimer structures. The best output structure was chosen considering the frequency of members inside each cluster and the Lowest Energy score.

## Results

### Membrane-Bound HLA-G1 Molecule Displays Tilting Motion in Solution

**Figure 2:**
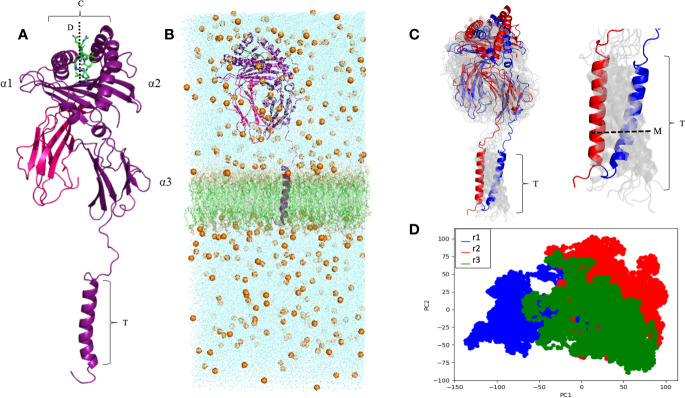
**(A)** Complete membrane-bound HLA-G1 protein encompassing the α1, α2, α3, transmembrane (T), and intracytoplasmic (I) domains (purple), coupled with β2-microglobulin (pink) and the RIIPRHLQL peptide (green). W (average cleft width) = 23.2 Å, and D (cleft depth) = 15.8 Å; T = 32.7 Å. **(B)** Complete membrane-bound HLA-G1 molecular dynamics simulation system, including: *i)* water molecules seen in the blue background, *ii)* Na^+^ and Cl^-^ ions (golden spheres), and *iii)* phospholipid bilayer (green). **(C)** Initial (blue) and final (red) conformations of the 100 ns complete membrane-bound HLA-G1 dynamics (left) and for the transmembrane portion (right). Intermediate conformations obtained at 10ns intervals are displayed in light gray, showing the molecular movement inside the lipid bilayer. M, transmembrane swinging movement over 100 ns. **(D)** Principal component analysis (PCA) depicting the distribution of conformations extracted from three independent MD trajectories (r1, r2, and r3).

A complete model of the mature protein encoded by the HLA-G*01:01 allele group was generated, containing all 314 residues (**Figure 2A**). The complete modeled system included the HLA-G molecule, sodium (Na^+^) and chloride (Cl^−^) counter-ions, and a phospholipid bilayer (**Figure 2B**). During the MD simulations, the average cleft width was 23.2 Å, ranging from 19.4 Å to 25.6 Å (measured at each 10 ns), and the peptide cleft depth was 15.8 Å (**Figure 2A**). The RIIPRHLQL peptide remained stable during the simulations, as observed by the low Root Mean Square Fluctuation (RMSF) (data not shown). The RMSD values for the MD simulations did not exceed 11.46 Å for any of the replicated trajectories, oscillating in the range from 4 Å to 10 Å (**Supplementary Figure 1**). Note that the observed RMSD variation does not reflect unfolding or large conformational changes in the protein, but it relates to oscillations on the angle of the transmembrane region and its impact on the orientation of the extracellular domain (**Figure 2C**). In fact, the RMSD value calculated between the initial and final conformations of the protein is of only 3.13 Å. This conformational stability can also be observed by the PCA analysis, which demonstrates great overlap of sampled conformations among all three simulations. Taken together, these results point to the stability and compactness of the complete membrane-bound HLA-G1 model generated (**Figure 2D**). The transmembrane region extended for 32.7 Å and, alongside the cytoplasmic tail, presented an all direction swinging movement, spanning 22.5 Å.

### ILT2 and ILT4 Interacting Residues Are Not Fully Accessible in the Membrane-Bound HLA-G1 Molecule

**Figure 3:**
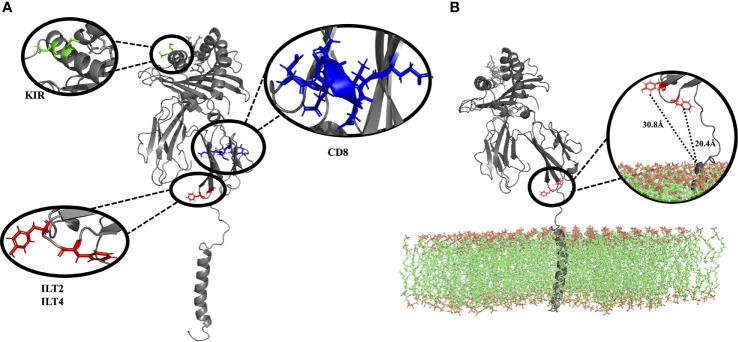
**(A)** Complete membrane-bound HLA-G1 protein (without lipid bilayer), indicating the interacting residues for CD8 receptor (blue: residues D223, Q224, T225, Q226, D 227, V228, E229), ILT2 receptor (red: residues F195), and ILT4 receptor (red: residues F195, Y197). Residues suggested to interact with KIR2DL are also depicted (green: residues Q79, M76) **(B)** Complete membrane bound HLA-G1 protein (including the lipid bilayer), emphasizing the localization and distance of the ILT2 (red: residues F195, 20.4 Å to the membrane) and ILT4 receptors (red: residues F195, Y197, 30.8 Å to the membrane), both of which are close to the lipid bilayer. (D, Aspartic acid; E, Glutamic acid; F, Phenylalanine; M, Methionine; Q, Glutamine; T, Threonine; and V, Valine).

According to previous studies, ILT2 binds to HLA-G residue F195, while ILT4 binds to F195 and Y197 (17, 38). All these residues are located at the end of the α3 domain, and our model shows that these binding sites are very close to the lipid bilayer (**Figure 3**). Limited access to these residues could explain the lower overall affinity of the HLA-G1 monomer to ILT2/ILT4, when compared to the soluble dimer, as previously demonstrated by *Shiroishi* and collaborators (17). Locations of other potential binding sites are also depicted. CD8α/α contacts the α3 domain of HLA-G1 at residues 223 to 229 (63, 64). Q79 and M76 are candidate interacting residues for KIR2DL4 (26, 65, 66).

### Soluble HLA-G1 Dimer Displays Full Rotational Freedom of Protomers

**Figure 4:**
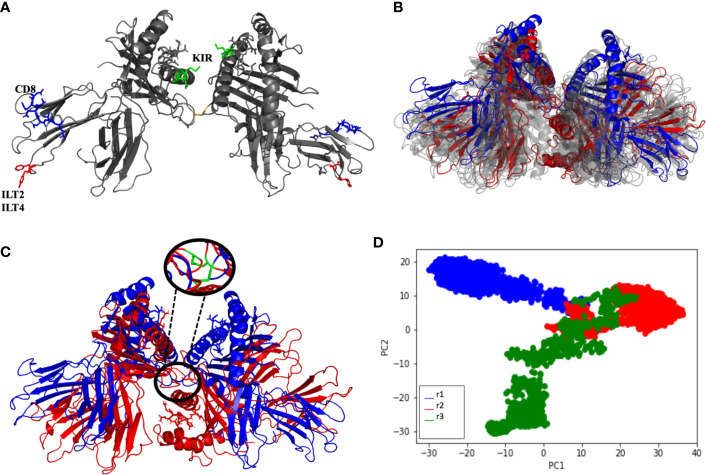
**(A)** Soluble HLA-G1 protein indicating the interacting for CD8 receptor (blue: residues D223, Q224, T225, Q226, D 227, V228, E229), ILT2 receptor (red: residue F195) and ILT4 receptor (red: residues F195, Y197). Residues suggested to interact with KIR2DL are also depicted (green: residues Q79, M76) **(B)** Initial (blue) and final (red) conformations of the 200 ns soluble HLA-G1 dimer dynamics. Twenty-nanosecond intervals (light gray) showing the significant dimer rotation. **(C)** Initial (blue) and final (red) conformations of the 200-ns soluble HLA-G1 dimer dynamics, depicting the zoomed area showing the disulfide bridge. **(D)** Principal component analysis (PCA) depicting the distribution of conformations extracted from three independent MD trajectories (r1, r2, and r3).

Three simulations of 200 ns were performed for the soluble HLA-G1 dimer, starting from the oblique orientation (~45° angle) observed by X-ray crystallography **(** **Figure 4A**) for the disulfide-linked HLA-G1 dimer. The RMSD values for the MD simulations oscillated between 9 Å and 25 Å, depending on the simulation (**Supplementary Figure 2**). Once again, these high RMSD values do not reflect conformational changes of the protomers (**Figures 4B, C**). Instead, they reflect the great conformational freedom of the protomers during the MD simulation, as enabled by the rotation of the disulfide bond (**Figure 4C**). Although the dimer as a whole is very flexible, the folding of the protomers is very stable, and the RIIPRHLQL peptide remained stably bound in the cleft; data consistent with the low Root Mean Square Fluctuation (RMSF) obtained (**Supplementary Figure 3**).

**Figure 5:**
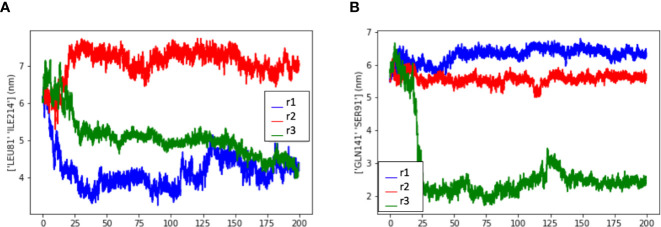
Measurement of the distance between residues to evaluate the dimer flexibility at the disulfide bridge. Measurement data extracted from three dimer simulations (r1, r2, and r3) showed that the residues exhibited similar spatial behavior in all simulations, depending on the residue-residue distance that is observed. **(A)** Measurement data from LEU81 and SER91, **(B)** Measurement data from LEU81 and ILE214. (LEU, Leucine; ILE, Isoleucine; GLN, Glutamine; SER, Serine).

Interestingly, the PCA analysis revealed that each soluble dimer simulation described a different trajectory, exploring different regions of the conformational space (**Figure 4D**). In our PCA analysis, PC1 is most correlated with the distance between LEU81 in one copy and ILE214 in the other copy (**Figure 5A**), while PC2 is most correlated with the distance between GLN141 in one copy and SER91 in the other copy (**Figure 5B**).

### HLA-G5 Is More Stable When Associated With β2-Microglobulin and a Peptide Ligand

**Figure 6:**
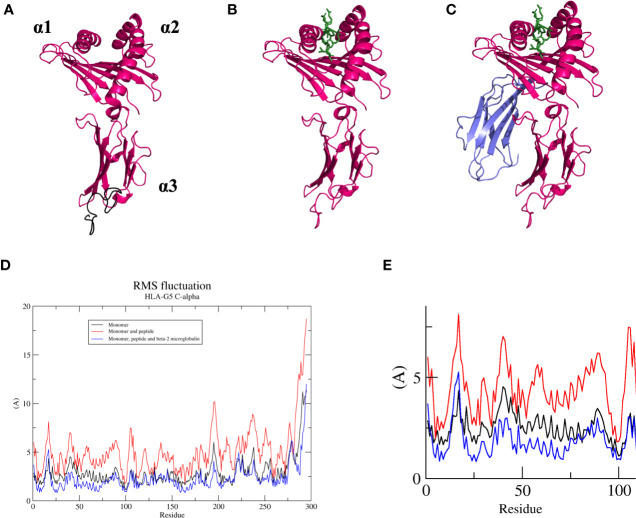
**(A)** Soluble HLA-G5 isoform (pink) and 21 amino acid tail from intron 4 (black, but represented in the following figures in pink). Domain location (α1, α2, and α3) shown. **(B)** Soluble HLA-G5 isoform (pink) and nonapeptide RIIPRHLQL (green). **(C)** Soluble HLA-G5 isoform (pink), nonapeptide RIIPRHLQL (green) and β2-microglobulin (lavender). **(D)** RMSF of all three HLA-G5 structural possibilities: monomer (black), monomer containing the nonapeptide in the cleft (red), and monomer containing the nonapeptide in the cleft coupled to β2-microglobulin (blue). **(E)** Zoomed α-1 domain residues (residue number 1–100), taken from RMSF plot **(D)**, showing HLA-G5 monomer (black), monomer containing the nonapeptide in the cleft (red), and monomer containing the nonapeptide in the cleft coupled to β2-microglobulin (blue).

**Figure 7:**
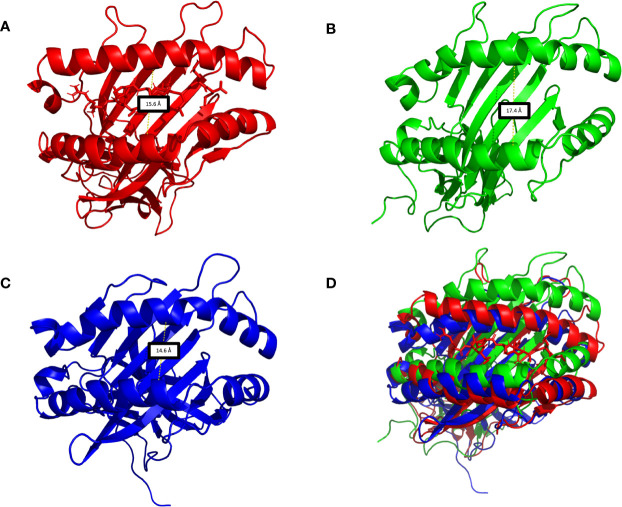
**(A)** HLA-G5 cleft in the absence of the β2-microglobulin, demonstrating the nonapeptide RIIPRHLQL in the initial moments of the simulations. **(B)** Around 200 ns of simulation, due to structural instability, the cleft widens and the nonapeptide RIIPRHLQL loses all interactions with the surrounding structures, escaping the cleft. **(C)** As there is no peptide left in the cleft, its width is diminished. **(D)** Superimposition of **Figures 6A–C**.

In this work, we evaluated three HLA-G5 structural possibilities: (*i)* monomer (**Figure 6A**), (*ii*) monomer containing the nonapeptide in the cleft (**Figure 6B** **)**, and (*iii*) monomer containing the nonapeptide in the cleft coupled to β2-microglobulin (**Figure 6C**). Considering Cα residue fluctuation of all the HLA-G5 structural possibilities, the most stable structure was the monomer containing the nonapeptide in the cleft coupled to β2-microglobulin, which suffered minimal structural deformations during the MD simulation **(** **Figure 6D** **)**. As seen in **(** **Supplementary Material**—HLA-G5 Monomer, nonapeptide, and coupled β2-microglobulin Simulation Video; **Supplementary Figure 4**), the stability is mainly due to the interaction of the tail from intron 4 and the coupled β2-microglobulin, which prevents the tail from reaching up and destabilizing the peptide cleft. In fact, this disruptive behavior was observed in the absence of β2-microglobulin, leading to complete dissociation of the nonapeptide from the HLA-G5 cleft (**Figure 6D** and **Supplementary Material**—HLA-G5 Monomer and nonapeptide Simulation Video). Specifically, the interaction with the tail from intron 4 (last 21 residues) resulted in an increase of the cleft’s width, causing the peptide’s anchor residues to lose important interactions with residues in the cleft’s β-sheet floor and surrounding α-helices (**Supplementary Figure 4**). At the beginning of the simulation the cleft width measured 15.6 Å (**Figure 7A**), increasing its size up to 17.4 Å around 200 ns of the simulation, when the peptide escapes the cleft (**Figure 7B**). The cleft width reduces to about 14.6 Å after the unbinding of the peptide (**Figure 7C**). The superimposed images reveal the variation in cleft’s width during the simulated time (**Figure 7D**).

Some structural instability was also observed for the soluble HLA-G5 monomer alone (**Figure 6D**). Both monomer and monomer containing the nonapeptide in the cleft showed much higher RMSF values for the α1-domain region, which constitutes residues 1 to 100. Such residues were extremely important in order to keep the peptide cleft folded, and suffered the majority of the destabilizing interactions induced by the movement of the portion relative to the tail from intron 4 (**Figure 6E** and **Supplementary Material**—HLA-G5 Monomer Simulation Video, **Supplementary Material**—HLA-G5 Monomer and nonapeptide Simulation Video).

### Produced Models Can Be Used for Additional Structural Analysis

All produced 3D models were made available through GitHub (github.com/KavrakiLab/hla-g-models) and can now be used as input for additional structural analysis. To demonstrate this point, we conducted a *(i)* peptide-docking analysis comparing two different HLA-G peptide-binders, and a *(ii)* protein-protein docking analysis of binding modes for ILT4.

Our structural analysis of the peptide-ligands indicated a similar overall contribution to complex stability. A structure-based machine learning method predicted a ~70% probability of stable binding for both peptides (**Supplementary Figure 5**). Moreover, the decomposition of the individual contributions of peptide residues indicated the dominant role of the conserved Leucine in p9 toward complex stability in both systems. As expected, there were differences in secondary interactions with other peptide’s residues, with a slight advantage toward RLPKDFRIL. Therefore, our analysis suggests that RLPKDFRIL would provide similar or slightly better stability to the tested HLA-G systems. This prediction is in agreement with recent experimental data showing no significant differences between these peptides regarding the binding of HLA-G1 to ILT2/ILT4 (67).

Our protein-protein docking analysis further corroborated the findings that better interaction with ILT4 is possible when using conformations of the soluble HLA-G1 dimer, as compared to the the membrane-bound HLA-G1 monomer. The putative ILT4-binding site is formed by a relatively hydrophobic patch formed by F195/Y197 residues. This is conserved in HLA-G molecules, but not in other classical HLAs (30). Indeed, our best HLA-G1 dimer/ILT4 interaction models are represented by hydrophobic-favored interactions involving these two HLA residues. Moreover, the ILT4 domains involved in this interaction were domains 1 and 2, which is in accordance to previous binding experiments (30) (**Supplementary Figure 6**). The best interaction model between the monomer of HLA-G and ILT4 was favored by electrostatic interactions and it is depicted in **Supplementary Figure 6A**. Note that the best results indicated a binding mode in which ILT4 approaches HLA-G1 from the “bottom” (**Supplementary Figure 6C**). This binding mode is different from that previously described by Wang et al. (67), and might only be possible for the soluble forms of HLA-G.

## Discussion

HLA-G plays an important role on the suppression of immune responses, and both membrane-bound and soluble isoforms may exert this function. As of September 2020, the IMGT-HLA database includes 80 *HLA-G* alleles, encoding 21 complete and 4 truncated proteins (*HLA-G*1*01:05N, G*01:13N, G*01:21N and G*01:25N) (68, 69). All alleles encoding the complete protein have the potential to *i)* form dimers through the conserved Cysteine at position 42, *ii)* form the seven commonly described HLA-G isoforms (HLA-G1 to HLA-G7), and *iii)* interact with the leukocyte receptors (26). This remarkable structural diversity must be studied in detail in order to clarify the diverse roles played by HLA-G molecules in both physiological and pathological conditions. Unfortunately, many questions remain unanswered about the structure, dynamics, expression and interaction patterns of different HLA-G alleles and isoforms. For instance, previous experimental studies have provided structures for the membrane-bound HLA-G1, and HLA-G1 dimer, either alone or interacting with other receptors. However, even in these cases the structures were incomplete. In addition, there was no available information on the dynamics of membrane-bound and soluble isoforms. Our goal was to conduct accurate structural modeling and molecular dynamics analysis of *i)* the complete membrane bound HLA-G1, *ii)* the soluble HLA-G1 dimer, and the soluble HLA-G5 monomer. This work moves the field forward, providing both insights on the dynamics of these complexes and complete 3D models that can now be used by other groups for further analysis.

Our complete model of HLA-G1 encompasses the heavy-chain (α1, α2, and α3 domains), connecting peptide, transmembrane portion and cytoplasmic tail of the most frequently observed HLA-G*01:01 molecule. The HLA-G*01:01 allele group encompasses 25 synonymous substitutions, as reported for the HLA-G*01:01:01:01 to HLA-G*01:01:25 alleles (68, 69). The associated light-chain (β2-microglobulin) was also included in our complete model. Finally, the RIIPRHLQL peptide, derived from histone H2A, was selected to be used in this study since (*i)* it is known to confer stability to the HLA-G molecule (64), (*ii)* is one of the most abundant peptides displayed by HLA-G (70), and (*iii)* was present in the cell cultures used for previous HLA-G X-ray diffraction studies (70). Note that an additional structural analysis comparing the binding of RIIPRHLQL with another HLA-G-binder, RLPKDFRIL, suggested that both peptides should provide similar level of stability to HLA-G complexes.

As expected, the MD simulations (**Figure 2B**) showed a stable membrane-bound HLA-G1 molecule, without evidence of unfolding of secondary structures (i.e., α-helices and β-sheets) (**Figure 2D**). In addition, both the β2-microglobulin and the coupled peptide ligand remained stably-bound during all simulations. Interestingly, we observed for the first time the natural “tilting” movement of the membrane-bound HLA-G1 in solution (**Supplementary Figure 1**). This motion, in addition to the lateral swinging movement of the transmembrane portion in the lipid bilayer (spanning 22.5 Å), is reflected on the observed RMSD values. However, the PCA analysis shows great agreement between simulations in terms of sampled conformations. The superposition of frames from the beginning and end of the simulation (**Figure 2D**) also shows that all secondary and tertiary structures were preserved during MD. These results confirms the stability and compactness of the complete membrane-bound HLA-G1 model generated (**Figure 2C**), which could now be used for additional structural analyses.

We also report for the first time the complete model of the soluble HLA-G1 dimer (**Figure 4A**), and the great conformational flexibility of this molecule in solution (**Figure 4B**). While the disulfide-linked dimer is locked into a 45° angle between the protomers (**Figure 1**), our simulations demonstrate that the soluble dimer is able to explore the full rotational flexibility enabled by the disulfide bridge (**Figure 4C**). Note that the secondary and tertiary structures of each protomer were very stable in solution (**Figure 4D**), despite overall dimer flexibility. The peptide-ligands also remained stably-bound to the respective clefts (**Figures 4A, B**). The PCA analysis of the three independent simulations of the soluble HLA-G1 dimer (**Figure 4B**) demonstrated that every dimer explored a different region of the conformational space, while still presenting similar collective motions, as demonstrated by the residue-residue distance comparisons (**Figures 5A, B**). A direct comparison between the PCAs for the membrane-bound HLA-G1 monomer and soluble HLA-G1 dimer is not possible, since the principal components used in each case reflect features that better capture the movements observed in each system. However, it is possible to observe that the soluble HLA-G1 dimer PCA captures a much greater conformational freedom.

Our HLA-G1 dimer model includes residues located at positions 195, 196, 197, 266, and 267, which were missing in the available crystal structure of the disulfide-linked dimer (17). All these residues are located in the α3 domain, where major leukocyte receptor binding sites are located. For instance, they include the putative binding sites for ILTs (residues 195 and 197) and CD8 (residues 223-229 residues). Note that these sites are very close to membrane in the membrane-bound HLA-G1 monomer (**Figure 3B**), which might limit interaction with these protein-ligands. It has indeed been observed that HLA-G1 dimers display higher affinity for leukocyte receptors than monomers (17, 20). This advantage has been associated with the 45° angle of the protomers in the disulfide-linked dimer, which can help exposing these sites for interaction (17, 67). Note that the free rotation of the protomers in the HLA-G1 soluble dimer, as observed in our simulations, would enable even greater exposure of these biding sites. In order to further explore our models and investigate the interaction with ILT4, we decided to conduct a protein-protein docking experiment with ClusPro. As expected, ClusPro successfully identified binding modes in which the D1 domain of ILT4 interacts with the α3 domain of HLA-G1, specifically involving F195 and Y197. Some of the predicted binding modes displayed ILT4 approaching HLA-G from the “top,” as previously described by Wang et al. (67). The authors of that study discuss the limited flexibility of ILT4 in terms of bending between Ig-like domains, and describe this “top-down” binding mode as the only interaction possible for membrane-bound forms of HLA-G1. Interestingly, in the absence of the membrane, ClusPro predicted better binding modes in which ILT4 approaches HLA-G1 from the “bottom,” while still preserving interactions between D1 and F195/Y197. Based on these results, we can speculate that higher affinity of soluble HLA-G1 dimer for ILT2/ILT4 ligands could be explained by the possibility of using this alternative “bottom-up” binding mode. Further computational and experimental studies would be necessary to investigate the occurrence and stability of alternative binding modes involving ILT2 and ITL4.

The interaction of HLA-G with T CD8^+^ cells may induce FasL up-regulation, soluble FasL secretion and CD8^+^ cell apoptosis by Fas-FasL interaction, whose binding sites have not been determined yet (71). Compared to classical class I molecules (e.g., HLA-A, -B, -C), HLA-G binds to CD8α/α loop (residues 223-229) with medium affinity (63, 64), thus inhibiting the T CD8^+^ cytotoxic function. Although little is known regarding the HLA-G dimer interaction with CD8, it is possible that the interaction confers increased avidity in a proper structural orientation, permitting an efficient signaling to CD8 as well as it does for ILT2/ILT4 (17, 38). The freedom of rotation reported in this study for the soluble HLA-G1 dimer, exposing two easily accessible binding sites for ILTs/CD8 receptors, corroborates the potential for multiple orientations of the dimer. Considering that these major leukocyte receptors are adjacent to each other, it is possible the formation of complexes containing multiple combinations of one HLA-G dimer and two leukocyte receptors (ILT2/ILT2, ILT4/ILT4; ILT2/ILT4, ILT2/CD8, ILT4/CD8, CD8/CD8).

It has been proposed that HLA-G could interact with the killer cell immunoglobulin-like receptor KIR2DL4 (25), and that such interaction could induce both inhibitory as well as activating signals (28, 72, 73). Although the inhibition of the innate and adaptive immune response is the most accepted role of HLA-G, activating responses have also been reported (74). The soluble form of HLA-G could be the natural KIR2DL4 ligand, since it accumulates in KIR2DL4^+^ endosomes and induces endosome signaling (75). In fact, structural representations (17) indicate that steric constraints would prevent KIR2DL4 from interacting with HLA-G dimers (65, 66, 75). Considering that KIR2DL binding residues are located in the α1 domain of the HLA-G molecule, and considering the HLA-G1 dimer rotation presented here, it is possible that the KIR2DL4 binding area would be more accessible in the soluble HLA-G1 dimer as opposed to the membrane-bound HLA-G1 dimer. However, it is important to stress that we have not tested this interaction in our study, and that recent studies have not found evidence of HLA-G/KIR2DL4 interaction. Once again, the models produced in this work can now be used to further investigate this potential interaction.

Contrary to what was observed for the aforementioned systems, greater instability was observed in two of our HLA-G5 models. Specifically, the only stable system was the one containing both the nonapeptide RIIPRHLQL and ß2-microglobulin **(** **Figure 6** and **Supplementary Material** – HLA-G5 Monomer, nonapeptide, and coupled β2-microglobulin Simulation Video). Previous studies have reported HLA-G5 isoforms both with and without β2-microglobulin (33, 76). However, our results suggest that the monomeric form of HLA-G5 would not be stable without these other chains (**Supplementary Material** – HLA-G5 Monomer Simulation Video). On the other hand, it is possible that HLA-G5 dimers could be stable in the β2-microglobulin-free form, which was not tested here. For instance, the intronic tails of both protomers could interact with each other, not causing the effect of cleft deformation observed in our simulations (**Supplementary Material** – HLA-G5 Monomer and nonapeptide Simulation Video). Such dimeric structures for HLA-G5 without β2-microglobulin could be similar to the dimers composed by the α1–α3: α1–α3 domains, as in the work published by Kuroki et al., in which HLA-G2 isoform (membrane bound α1–α3) naturally formed a β2-microglobulin-free homodimer which did not have disulfide bridges keeping the structures in place (77). Electron microscopy revealed that the general structure and domain organization of such HLA-G2 homodimers resembled those of class II HLA heterodimers (α1–α2: β1–β2) (20). Published data (77) described the binding of β2-microglobulin and β2-microglobulin-free forms of HLA molecules to members of ILT receptor family and demonstrated that, in addition to ILT4, β2-microglobulin-free structures are recognized by several other members of this receptor family. In fact, the “activating” members of the ILT family showed a preference for such structures. Therefore, it is possible that this could also be the case for HLA-G. This would support the notion that structural variations of HLA-G may be relevant in the modulation of biological function (32). It’s also intriguing to consider that, similar to classic class I HLA molecules, HLA-G may have activating receptors. In fact, it is possible that there are other receptors for HLA-G, specific for isoforms or not, and the study of HLA-G structures other than HLA-G1 and HLA-G5 may allow us to identify them (32).

In conclusion, the present study describes for the first time the complete membrane-bound HLA-G1 3D structure and its dynamic behavior in solution. Our study also described the dynamics of the soluble HLA-G1 dimer. Our simulations highlighted the great flexibility enabled by the disulfide bridge, which could even promote alternative binding modes with ILT2/ILT4 receptors. Our study of the HLA-G5 isoform and its structural alternatives demonstrated greater structural instability when the peptide or β2-microglobulin were absent. More comprehensive structural studies will be necessary to verify the existence of other structural conformations for HLA-G5. This work produced insights on the structure, dynamics, and interaction patterns of important HLA-G variants. It also produced 3D models that can now be used to further investigate these and other HLA-G molecules, to identify new HLA-G ligands, and to design potential pharmacological interventions.

## Data Availability Statement

The datasets presented in this study can be found in online repositories. The names of the repository/repositories and accession number(s) can be found in the article/**Supplementary Material**.

## Author Contributions

TA, DA and ED contributed to the conception and design of the study. TA generated all structural models. TA and DA performed simulations. TA, DA, MR and JA performed the analysis. SG and LK supervised the analysis. TA, DA and ED wrote the article. All authors contributed to the article and approved the submitted version.

## Funding

This work was supported in part by Coordenação de Aperfeiçoamento de Pessoal de Nível Superior (CAPES). This work was also partially supported by the Cancer Prevention & Research Institute of Texas (CPRIT), through award number RP170508, and through a fellowship from the Gulf Cost Consortia on the Computational Cancer Biology Training Program (grant number RP170593). Finally, this work was also partially supported by a training fellowship from the National Library of Medicine Training Program in Biomedical Informatics (grant number T15LM007093), and by Rice University funds.

## Conflict of Interest

The authors declare that the research was conducted in the absence of any commercial or financial relationships that could be construed as a potential conflict of interest.
