# Supplementary material for: Structural Modeling and Molecular Dynamics of the Immune Checkpoint Molecule HLA-G
Source: Front Immunol. 2020 Nov 6;11:575076. doi: 10.3389/fimmu.2020.575076 (PMC7677236; doi:10.3389/fimmu.2020.575076)
Supplement: Supplementary file 1 [file DataSheet_1.pdf]

Supplementary Material

1 Supplementary Data

All structures and simulation movies are available in the Supplementary Material and at GitHub (https://github.com/KavrakiLab/).

- Membrane-bound HLA-G1 PDB model (file Membrane-bound_HLA-G1.pdb).
- HLA-G1 Dimer PDB model (file HLA-G1_Dimer.pdb).
- HLA-G5 Monomer PDB model (file HLA-G5_monomer.pdb).
- HLA-G5 Monomer and nonapeptide PDB model (file HLA-G5_monomer_peptide.pdb).
- HLA-G5 Monomer, nonapeptide and coupled β2-microglobulin PDB model (file HLA-G5_pep_beta.pdb).
- HLA-G5 Monomer Simulation Video (file G5_monomer_r1.mpg).
- HLA-G5 Monomer and nonapeptide Simulation Video (file G5_pep_r1.mpg).
- HLA-G5 Monomer, nonapeptide and coupled β2-microglobulin Simulation Video (file G5_pep_beta_r1.mpg).
- ClusPro ILT4-HLA-G1 Monomer Protein-Protein docking model (file ClusPro_ILT4-HLA-G1_monomer.pdb).
- ClusPro ILT4-HLA-G1 Soluble Dimer Protein-Protein docking model (file ClusPro_ILT4-HLA-G1_soluble-dimer.pdb).
- APE-Gen RIIPRHLQL-HLAG-1 Peptide-docking model (file ApeGen_RIIPRHLQL-HLAG1_openmm-em.pdb).
- APE-Gen RLPKDFRIL-HLAG-1 Peptide-docking model (file ApeGen_RLPKDFRIL-HLAG1_openmm-em.pdb).

2 Supplementary Table 1. HLA-G structures available on the Protein Data Bank (PDB, https://www.rcsb.org).

PDB_ID	Sequence Length (aa)	Peptide	Resolution (Å)	Isoform	Monomer/Dimer	Membrane-bound /Soluble
**1YDP**	275	RIIPRHLQL	1.90	HLA-G1	Monomer	Soluble
**2D31**	276	RIIPRHLQL	2.30	HLAG-1	Dimer	Soluble
**2DYP**	277	RIIPRHLQL	2.50	HLAG-1	Monomer	Soluble
**3KYN**	275	KGPPAALTL	2.40	HLAG-1	Monomer	Soluble
**3KYO**	273	KLPAQFYIL	1.70	HLAG-1	Monomer	Soluble
**6AEE**	277	RIIPRHLQL	3.30	HLAG-1	Monomer	Soluble
**6K60**	277	RIIPRHLQL	3.15	HLAG-1	Monomer	Soluble

3 Supplementary Figures

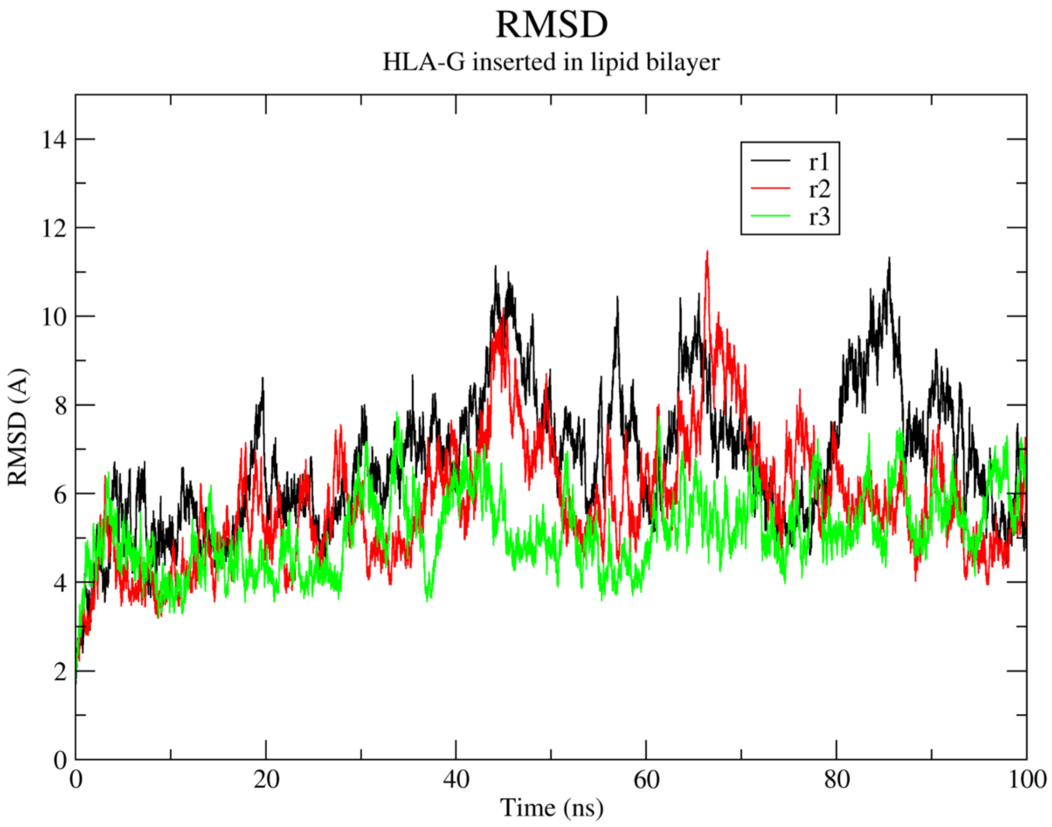

Supplementary Figure 1. Root Mean Square Deviation (RMSD) graph for all heavy-atoms in the system. Each line indicates an independent simulation (r1, r2, r3) of the membrane-bound HLA-G1 inserted into a phospholipid bilayer.

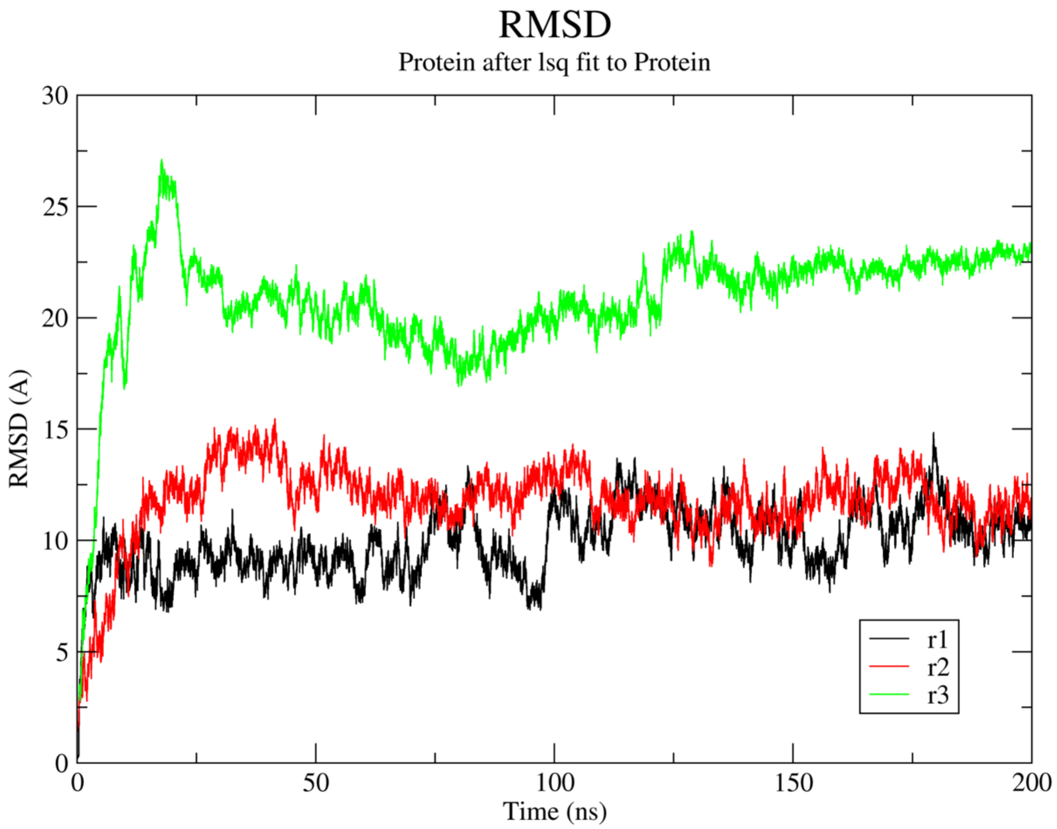

Supplementary Figure 2. Root Mean Square Deviation (RMSD) graph for all heavy-atoms in the system. Each line indicates an independent simulation (r1, r2, r3) of the soluble HLA-G1 dimer.

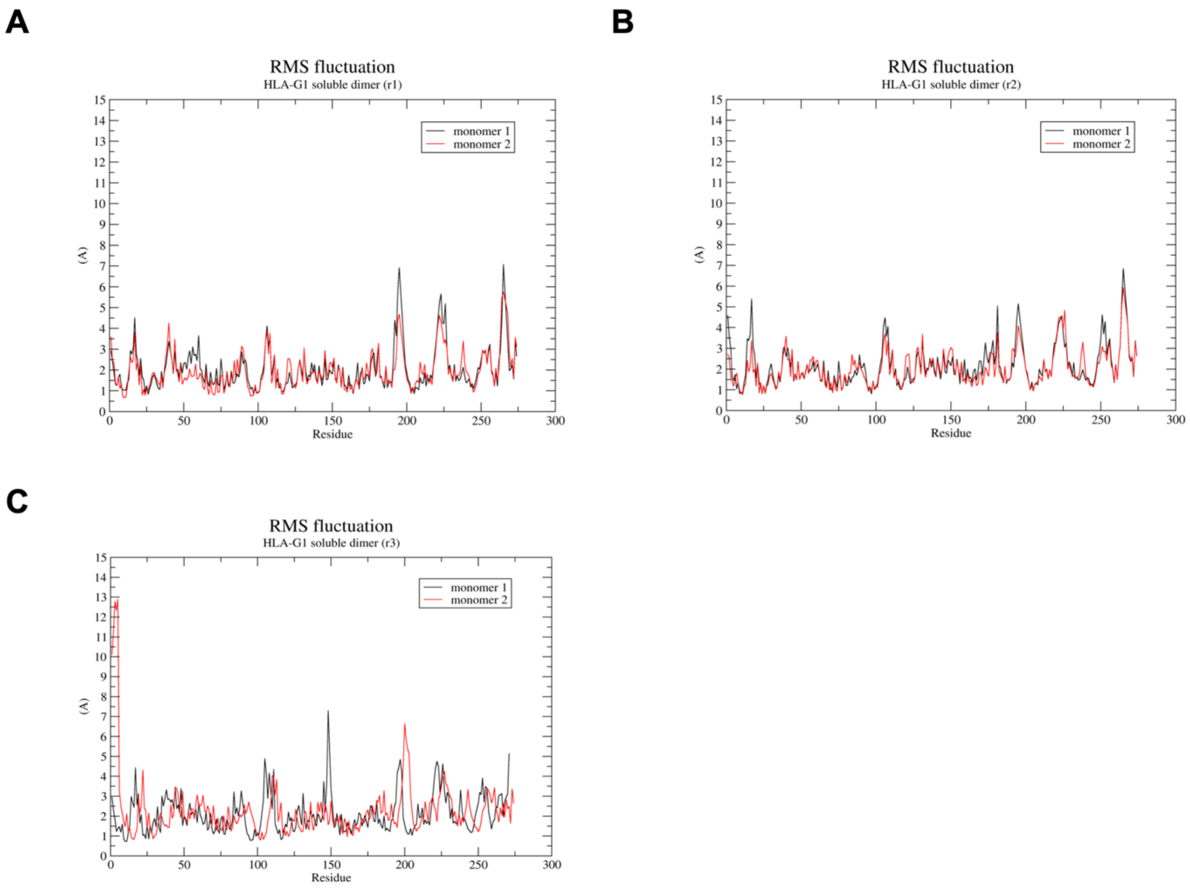

Supplementary Figure 3. Root Mean Square Fluctuations (RMSF) for the three HLA-G1 soluble dimer simulations: r1 (**A**), r2 (**B**) and r3 (**C**).

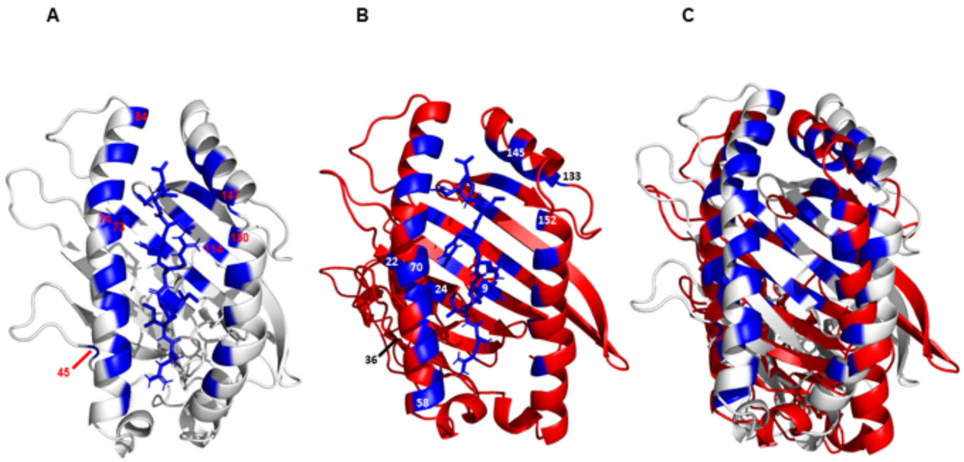

Supplementary Figure 4. HLA-G5 isoform containing the nonapeptide in the cleft formed by the α1 and α2 helices in the initial (A, in white) and final (B, in red) moments of the 300 ns MD simulation. **(A)** Top view of the HLA-G5 isoform, showing in the foreground the cleft formed between the α1 and α2 domain, and the peptide. This figure demonstrates the interactions of the peptide residues with the cleft during the initial moments of the MD simulation. The unique residues for the initial moments are numbered. **(B)** Top view of the HLA-G5 isoform, showing in the foreground the cleft formed between the α1 and α2 domain, and the peptide. This figure demonstrates the interactions of the peptide residues with the groove during the final moments of the MD simulation. The unique residues for the final moments are numbered. **(C)** Comparison of peptide residues interactions with the cleft in the initial (white) and final (red) moments (peptide not shown for better observation of the β-sheets).

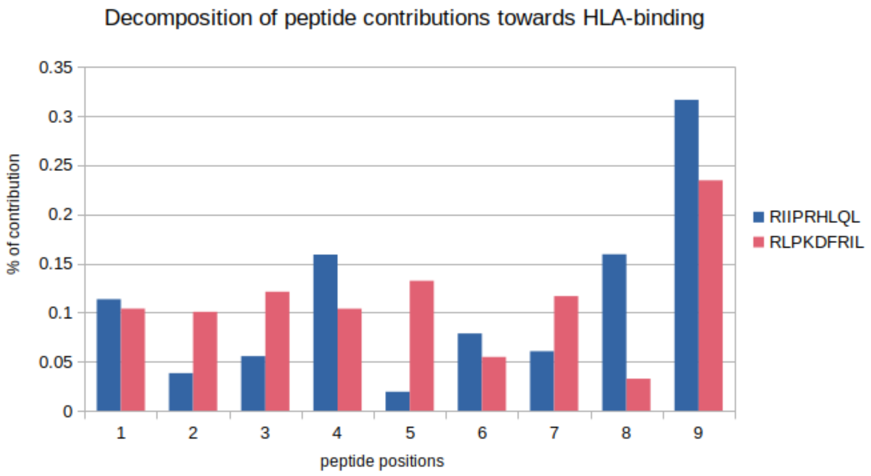

Supplementary Figure 5. Decomposition of peptide's residue contributions towards HLA-binding and peptide-HLA stability, according to a structure-based random forest predictor. This method was previously trained and validated using immunopeptidomics data. Using as input 3D models produced by APE-Gen for two peptides of interest (RIIPRHLQL and RLPKDFRIL), the random forest method predicts a ~70% probability of stable binding for both peptides. The individual contributions of peptide residues indicated the dominant role of the conserved Leucine in p9 towards complex stability in both systems.

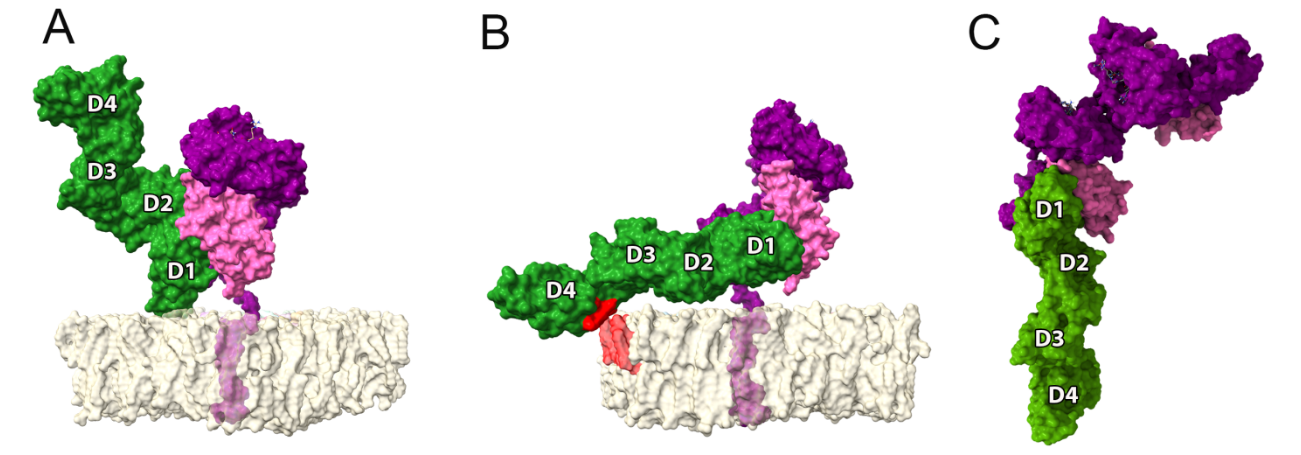

Supplementary Figure 6. Protein-protein docking analysis with ClusPro. **(A)** "Top-down" binding mode between membrane-bound HLA-G1 monomer (purple and pink) and ILT4 (green). This binding mode has been described in previous crystallographic studies, and was reproduced among the binding modes predicted by ClusPro. **(B)** Best binding mode predicted by ClusPro for the membrane-bound HLA-G1 monomer shows a "bottom-up" interaction. The membrane structure was not included in ClusPro calculations. By overlaying the ClusPro result with the membrane structure it becomes clear that this binding mode would not be possible in the presence of the membrane, as highlighted by the clashing atoms (red). **(C)** The best predicted binding mode for the soluble HLA-G dimer (purple and pink) and the ILT4 (green) also depicted this "bottom-up" interaction. In all cases, HLA-G interactions were mediated by ILT4 D1 and D2 domains.
